# Decadal Changes in Institutional Diagnostic Reference Levels for X-Ray Angiography: A Retrospective Comparative Study

**DOI:** 10.3390/jimaging12070333

**Published:** 2026-07-22

**Authors:** Ioannis Antonakos, Emmanouil Anousis, Tatiana Roko, Antonia Alexiadou, Maria Dimitropoulou, Dimitris Filippiadis, Stavros Spiliopoulos, Konstantinos Palialexis, Athanasios Giannakis, Niki Parmenidou, Efstathios Efstathopoulos

**Affiliations:** 1 Department of Applied Medical Physics, Medical School, National and Kapodistrian University of Athens, 12462 Athens, Greece; emmanouil.annousis@gmail.com (E.A.); rokotatiana@gmail.com (T.R.); alexiadou.antonia@gmail.com (A.A.); mdimitropoulou02@gmail.com (M.D.); 2 2nd Department of Radiology, National and Kapodistrian University of Athens, Chaidari Athens, 12461 Athens, Greece; dfilippiadis@med.uoa.gr (D.F.); stavspiliop@med.uoa.gr (S.S.); kpalialex@gmail.com (K.P.); a-giannakis@hotmail.com (A.G.); niki.parmenidou@gmail.com (N.P.); stathise@med.uoa.gr (E.E.)

**Keywords:** angiography procedures, Diagnostic Reference Levels, interventional radiology, radiation protection, fluoroscopy, dose area product

## Abstract

Angiography is a key imaging modality for the diagnosis and treatment of vascular diseases, and the growing sophistication of interventional procedures has heightened the need for radiation dose optimization. Diagnostic Reference Levels (DRLs) are widely used to monitor patient exposure and to support optimization in accordance with the ALARA principle. This study compared radiation dose metrics from a newly installed angiographic system at Attikon University Hospital with those obtained from the institution’s previous system and with values reported in the published literature. Radiation dose and procedural parameters were retrospectively collected for digital cerebral subtraction angiography (DSA), embolization, nephrostomy, vertebroplasty, transjugular intrahepatic portosystemic shunt (TIPS), chemoembolization, and injection procedures. Dose area product (DAP), fluoroscopy-related DAP, patient entrance dose indicators, and fluoroscopy time were analyzed. Median DAP values ranged from 5.72 to 349.60 Gy·cm^2^ depending on the procedure. Compared with data acquired approximately a decade earlier, DAP values increased by an average of 96.4%, whereas fluoroscopy times remained largely unchanged. Despite this increase, dose levels were generally lower than those reported in the international literature. Median DAP values ranged from 5.72 to 349.60 Gy·cm^2^ depending on the procedure. Compared with data acquired approximately a decade earlier, DAP values increased by an average of 96.4%, whereas fluoroscopy times remained largely unchanged. Although these differences may reflect the combined influence of technological developments, evolving procedural complexity, operator-related factors, and changes in clinical practice over time, these variables were not directly assessed in the present retrospective study. Nevertheless, the updated institutional Diagnostic Reference Levels provide a valuable benchmark for radiation dose optimization, quality assurance, and future multicenter studies aimed at supporting national DRL establishment.

## 1. Introduction

Digital subtraction angiography (DSA) is a fluoroscopic imaging technique used to visualize blood vessels and detect vascular abnormalities. It operates by digitally subtracting a pre-contrast “mask” image from post-contrast images acquired after contrast administration, thereby removing overlapping structures such as bone and soft tissue and enabling clear visualization of vascular anatomy, particularly in small and peripheral vessels [1,2]. DSA is widely regarded as the gold standard for evaluating many vascular diseases, particularly those affecting the cerebrovascular system and peripheral arteries. It provides superior spatial resolution compared with computed tomography angiography (CTA) and magnetic resonance angiography (MRA), and it is widely used to guide endovascular procedures such as thrombectomy, stenting, and embolization [2,3]. In addition, DSA plays an important role in the assessment of complex vascular disorders and in treatment planning when non-invasive imaging is insufficient [3,4]. Angiographic techniques are also applied in a range of interventional procedures, including nephrostomy, percutaneous interventions, vertebroplasty, and transjugular intrahepatic portosystemic shunt (TIPS) procedures [5,6,7].

The increasing complexity and frequency of angiographic procedures have highlighted the need for standardized tools to monitor and optimize radiation exposure for both patients and clinical staff [8,9]. In this context, Diagnostic Reference Levels (DRLs), introduced by the International Commission on Radiological Protection (ICRP) and the European Commission, serve as a key tool for optimization of radiation dose in medical imaging. Initially defined in ICRP Publication 73 (1996) as investigation levels for identifying unusually high patient doses, DRLs were further reinforced in Publication 135 (2017) as an essential component of radiation protection in diagnostic and interventional radiology. DRLs are not dose limits and may be exceeded when clinically justified. They are defined for groups of patients rather than individuals and may be established at local, regional, or national levels [10,11,12,13,14].

Accordingly, DRLs act as reference values for dose optimization and quality assurance in accordance with the ALARA (As Low As Reasonably Achievable) principle. They facilitate the identification of typical radiation dose levels across procedures, equipment, and protocols, with exceedances prompting review and optimization. Regular updating of DRLs supports continuous improvement in clinical practice and dose reduction, while also enabling inter-institutional comparison of radiation exposure [10]. Although Diagnostic Reference Levels (DRLs) are an important tool for optimizing patient radiation protection, there are still methodological difficulties in how they are defined and applied. These challenges are especially significant in interventional diagnostic and therapeutic procedures, where procedural complexity can vary widely. For simple X-ray exams using film-screen systems, differences in radiation dose are mainly caused by patient size, while differences in how operators perform the exam are usually small when standard protocols are followed. However, in more complex procedures that involve significant fluoroscopy, dose variation is influenced much more by the operator’s decisions and the difficulty of each case [15]. To improve consistency and comparability, it is recommended to standardize procedural nomenclature and to establish separate DRLs for diagnostic and therapeutic procedures [16].

Consistent with European guidelines, DRLs are typically based on the 75th percentile (third quartile) of the dose distribution [13]. As outlined in European Commission Radiation Protection No. 185, local DRLs are established using data collected within a single large institution or across multiple facilities for a defined clinical imaging procedure [11]. In interventional radiology practice, DRLs are commonly expressed using dose indicators such as dose–area product (DAP), cumulative reference point dose, fluoroscopy time, and fluoroscopic DAP [8,10].

Despite the widespread use of DRLs in interventional radiology, there remains a need for updated institution-specific reference levels that reflect contemporary angiographic equipment and current clinical practice. Therefore, the aim of this study was to establish updated local DRLs for a range of interventional radiology procedures performed using a Philips Azurion 7 angiography system (Philips, Amsterdam, The Netherlands) at Attikon University General Hospital. These values were compared with those obtained approximately ten years earlier using the institution’s previous Toshiba INFX-8000V (Toshiba Medical Systems Corporation, Otawara, Tochigi, Japan) angiography system and with published DRLs reported in the international literature. Because of the retrospective nature of the study, the comparison should be interpreted as reflecting changes occurring over the study period rather than the isolated effect of the imaging equipment.

The primary objective was not only to update local DRLs but also to evaluate how radiation exposure metrics have evolved over approximately one decade of routine clinical practice within the same institution.

## 2. Materials and Methods

### 2.1. Study Design

This retrospective study was conducted at Attikon University General Hospital and included procedures performed during 2016–2017 using INFX-8000V—Type S (Toshiba Medical Systems Corporation, Otawara, Tochigi, Japan) delivered by Toshiba and their counterparts, and in Azurion 7 B20/15 developed by Philips, throughout the period 2025–2026. A total of 562 interventional radiology procedures were included in the analysis. The distribution of procedures between the two angiography systems is presented in Table 1. Sex distribution was comparable between the two cohorts, with female patients accounting for 52.4% and 51.3% of procedures performed using the Toshiba and Philips systems, respectively. Patient age, body mass index, and other anthropometric characteristics were not consistently available in the retrospective database and could therefore not be incorporated into the analysis. Consequently, potential differences in patient characteristics between the two study periods could not be evaluated and should be considered when interpreting the results.

According to the literature, newer and more advanced angiography systems are associated with reduced DAP [17]. Technical specifications for both of the systems are mentioned in Table 2. Patient IDs were used to ensure privacy and to comply with personal data regulations. Also, patient selection was not subject to any specific criteria, in order to ensure that the establishment of local DRLs is not partial.

Detector performance was additionally characterized using Detective Quantum Efficiency (DQE), which describes the efficiency with which an imaging detector converts incoming X-ray photons into useful image information while preserving image quality. Higher DQE values generally indicate improved detector performance and may contribute to dose optimization by maintaining image quality at lower radiation exposures.

### 2.2. Interventional Procedures

Procedures were performed using the respective flat-panel angiography system equipped with automatic dose rate control, which also selects a suitable fluoroscopy pulse rate. The exposure parameters were set manually by radiologists.

The interventional procedures under study are cerebral DSAs (Digital Subtraction Angiographies), embolization for hemorrhages, nephrostomies, vertebroplasties, percutaneous procedures, TIPSs (Transjugular Intrahepatic Portosystemic Shunts), TACEs (Transarterial Chemoembolizations) and injections. Discrimination criteria for these interventional procedures revolve around clinical urgency, coagulation status (INR/platelets), contrast contraindications, and anatomical goals. Guidelines are standardized by medical bodies like the Society of Interventional Radiology (SIR) and the American College of Radiology (ACR).

### 2.3. Radiation Dose Metrics

Radiation dose metrics include Dose Area Product (DAP), dose at Reference Point (dose RP), fluoroscopy DAP and total fluoroscopy time. These data were all extracted from the angiography system dose reports within Paxera Ultima.

DAP represents the product of the dose at the center of a certain surface of the X-ray beam (e.g., the surface of the patient) multiplied by the area of the X-ray field at that surface. This is an extremely important quantity because it remains constant due to the fact that the dose decreases with the square of the distance, while the surface area increases with the square of the distance [15]. Thus, it corresponds to the estimation of stochastic risk but is not directly useful for estimating tissue reactions. DAP is an important indicator of the overall radiation burden and is closely associated with the stochastic risk to the patient. However, it does not directly reflect the maximum absorbed skin dose and therefore cannot be used alone to assess the risk of deterministic radiation injuries. For this purpose, the cumulative dose at the patient entrance reference point provides a more appropriate indicator.

Dose at a defined reference point can be used to estimate peak skin dose and therefore the risk of deterministic injuries. For an iso-centric interventional fluoroscope, the reference point is usually located 15 cm from the isocenter toward the X-ray tube, known as the patient entrance reference point [20,21]. The reference point moves with the gantry in such systems.

Fluoroscopy DAP is the partial DAP that was generated genuinely during fluoroscopy, and fluoroscopy time is the total accumulated time duration during which real-time X-ray imaging was used. Nevertheless, the last one is a poor indicator of exact patient dose because it doesn’t take into consideration significant parameters such as changes in field size, dose rate, or collimation [22].

### 2.4. Statistical Analysis

Statistical analyses were performed using SPSS version 29. For each procedure, descriptive statistics including the median, first quartile (Q1), third quartile (Q3), and interquartile range (IQR) were calculated separately for each angiography system. In accordance with European recommendations, the third quartile (75th percentile) was considered the proposed local Diagnostic Reference Level. Median values were additionally reported to facilitate comparison of the central tendency between the two study periods and to provide a robust summary of the dose distributions. Box plots were generated for DAP and fluoroscopy time, while pie charts were used to illustrate the relative contribution of fluoroscopy DAP to total DAP. Differences between the two angiography systems were further evaluated using the Mann–Whitney U test because the data were not normally distributed.

## 3. Results

This research compared invasive procedures performed using the new angiography system Philips Azurion 7, with their counterparts performed ten years earlier using the old system, Toshiba INFX-8000V. The comparison was based on Diagnostic Reference Levels (DRLs) and the median values of Dose Area Product (DAP) obtained from two angiography systems. The results are presented in Table 3 along with additional descriptive statistics, including the mean, the 25th and 75th percentiles, and the interquartile range (IQR) for each procedure and for each angiography system. As shown in Table 3, the median DAP values for the new angiography system were higher than those of the older system for all procedures except DSA cerebral examinations, in which a significant reduction has been achieved and vertebroplasty, where values are roughly at the same level.

In contrast, the median values of total dose at Reference Point (RP) were found to be lower in the new angiography system for several procedures, including DSA cerebral examinations, embolization, nephrostomy, and vertebroplasty. However, higher median doses at RP were observed for TIPS, TACE, and injection procedures. The median values and all other descriptive statistics evaluated in this study are presented in Table 4.

In addition to dosimetry parameters, data related to the total fluoroscopy time were collected. For the majority of procedures, the total fluoroscopy time was similar between the two angiography systems. However, a notable reduction was observed in DSA cerebral procedures, whereas increased fluoroscopy times were observed in procedures such as TIPS and TACE, as presented in Table 5.

**Figure 1:**
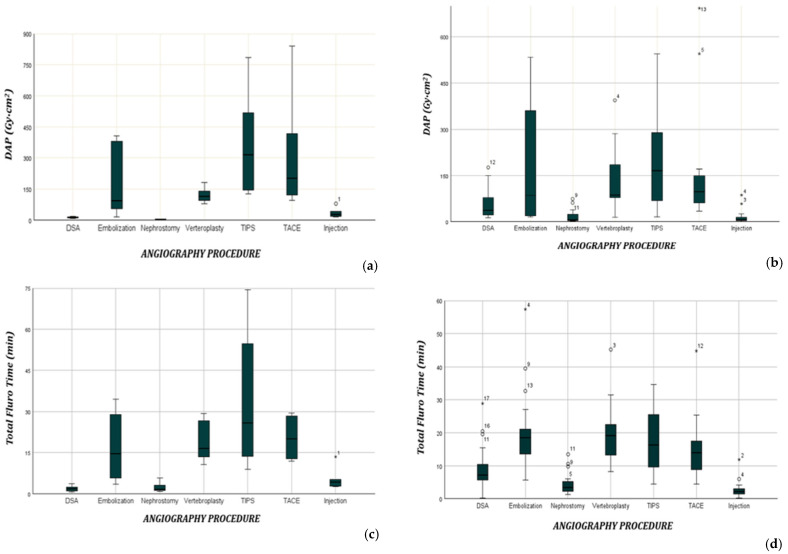
Box plots (**a**): DAP for each procedure operated at the new angiography system Philips Azurion 7; (**b**): DAP for each procedure operated at the old angiography system Toshiba INFX-8000V—Type S; (**c**): fluoroscopy time for each procedure operated at the new angiography system Philips Azurion 7; (**d**): fluoroscopy time for each procedure operated at the old angiography system Toshiba INFX-8000V—Type S.

In addition to descriptive statistics presented in tables, boxplots for both DAP and fluoroscopy time were generated to provide a visual comparison. Figure 1 presents DAP boxplots for the new and old angiography systems, respectively. The corresponding boxplots for total fluoroscopy time are shown in Figure 1.

After performing the normality tests and confirming that the data were not normally distributed, the non-parametric Mann–Whitney U test was used to evaluate whether differences in DAP and total fluoroscopy time were statistically significant between the two angiography systems. The results indicated statistically significant differences in both DAP and fluoroscopy time in three procedures: DSA cerebral, embolization and injections (*p* < 0.05). For these procedures, the Mann–Whitney U test revealed a statistically significant difference in DAP between the two systems, with a moderate to large effect size. Regarding fluoroscopy time, similar findings were observed, with the exception of the injection procedure, where a statistically significant difference between the two systems was identified, with a small to moderate effect size. All relevant statistical results are presented in Table 6.

**Figure 2:**
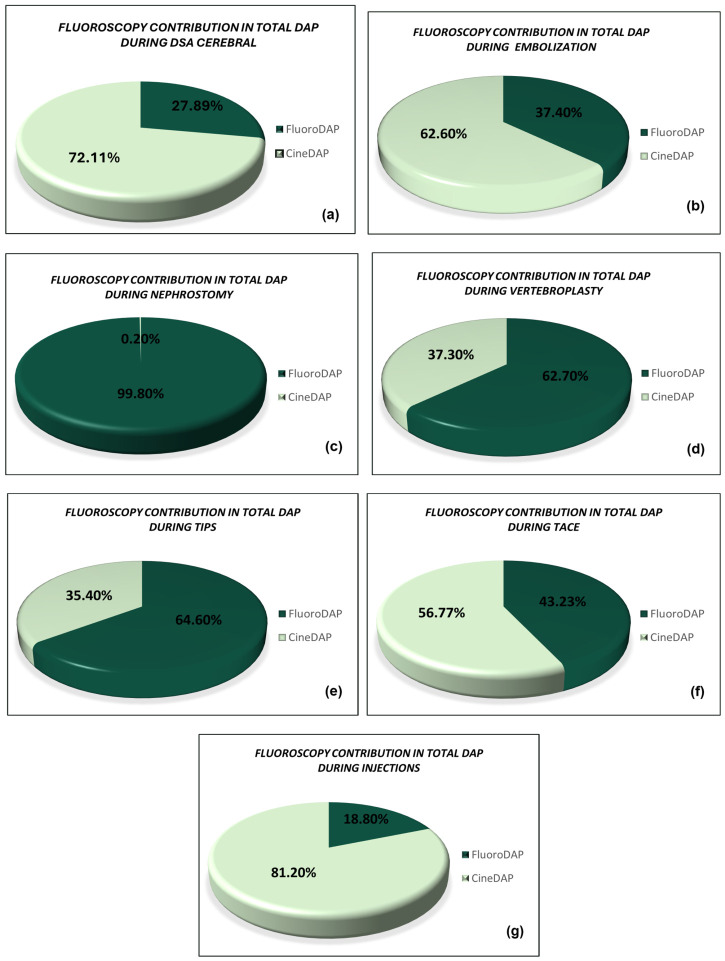
Pie charts about fluoroscopy DAP contribution in total DAP for each procedure: (**a**) DSA cerebral, (**b**) embolization, (**c**) nephrostomy, (**d**) vertebroplasty, (**e**) TIPS, (**f**) TACE and (**g**) injections.

Because the new angiography system also provides fluoroscopic Dose Area Product (DAP), the contribution of fluoroscopy to the total DAP was calculated. Since the Mann–Whitney U test does not directly provide information on the percentage differences in the parameters between the two units, the percentage relative differences were computed for DAP (DSA: −55.1%, embolization: +132,6%, injections: +173.3%) and time (DSA: −69.8%, embolization: −8.1%, injections: +32.3%). The results are presented in Figure 2 as pie charts for each procedure. As we observe from the pies in three procedures: nephrostomy, vertebroplasty and TIPS, the contribution of fluoroscopy was higher than 62%.

## 4. Discussion

This study re-evaluated institutional Diagnostic Reference Levels (DRLs) for a range of interventional radiology procedures by comparing radiation exposure metrics collected approximately one decade apart within the same tertiary hospital. The comparison provides insight into how radiation dose indicators have evolved over time under routine clinical practice while minimizing variability associated with differences between institutions. Although the introduction of a newer angiography platform represents an important component of this evolution, the observed differences should be interpreted within the broader context of changes in procedural complexity, operator-related factors, and clinical practice that may also have occurred during the study period.

To be precise, for TIPS, TACE, and injection interventions, the observed increase in procedure duration justifies the higher dose at the reference point and DAP. Increased operator experience, greater familiarity with guidance techniques, and the gradual standardization of procedures may have contributed to the reduction in fluoroscopy duration [23]. However, it is not entirely attributable to this rise, as these parameters also depend on other factors, such as the field size, the acquisition duration and protocol, the pulse and frame rate, the magnification used, collimation, and dose distribution [22,23]. Similarly, for cerebral DSA, a reduction in fluoroscopy time indicates a reduction in DAP and dose RP, as observed. Regarding hemorrhage embolization, nephrostomy replacement, and vertebroplasty procedures, although a reduction in time and dose is observed at the reference point, the total DAP increases. Statistically significant differences were found for DAP and total fluoroscopy time during DSA cerebral procedures (−55.1% and −69.8% for DAP and time, respectively), embolization (+132.6% and −8.1%), and injections (+173.3% and +32.3%). The observed differences in DAP, dose at the reference point, and fluoroscopy time are likely multifactorial. Besides technological developments, radiation exposure during interventional procedures is influenced by patient-related characteristics, procedural complexity, operator experience, imaging protocols, fluoroscopy settings, acquisition techniques, and clinical decision-making. Because these variables were not systematically recorded in the present retrospective study, their individual contribution cannot be quantified. Consequently, the observed differences should not be interpreted as the isolated effect of the angiography system but rather as the combined result of technological evolution and changes in clinical practice over the approximately ten-year study interval. This interpretation is consistent with current recommendations emphasizing that radiation dose metrics in interventional radiology are determined by multiple interacting factors rather than equipment characteristics alone.

A comparison of the results of this study with the available data in the international literature showed that the recorded radiation exposure levels generally fall within the ranges reported for similar interventional radiological procedures, though they vary depending on the type and complexity of the procedure. A comparison with published data for DSA procedures is presented in Table 7. DAP and Dose RP values in this study were lower than those reported by Slare et al. and Opitz et al., while they remained comparable to the results of Papanastasiou et al. and Actan et al. Furthermore, the recorded fluoroscopy time was significantly shorter compared to most published studies, a finding that may reflect optimized workflows and more efficient management of the procedure.

Table 8 summarizes the comparison of embolization dose metrics with previously published studies. The DAP value recorded in this study was higher than in most published reports, yet remained lower than the levels described by Actan et al. In contrast, the Dose RP values and fluoroscopy time were significantly lower compared to several previous studies, suggesting that the increased total DAP value is likely not exclusively due to the duration of fluoroscopy but may be related to factors such as the size of the irradiation field, acquisition protocols, or the use of cine acquisitions [9,25].

Table 9 presents a comparison of the recorded TIPS dose metrics with corresponding values reported in the literature. DAP, Dose RP and fluoroscopy time values were comparable to those reported by Pimenta et al. and Compagnone et al. The high dose observed in these procedures is expected, given the increased technical difficulty, the need for multiple angiographic views, and prolonged fluoroscopic guidance.

The comparison of TACE radiation exposure parameters with published reference values is presented in Table 10. Specifically, DAP was lower compared to the studies by Pimenta et al., RP 195, Rizk et al., Etard et al., and Ruiz-Cruces et al., while only the study by Compagnone et al. reported a lower value. A similar trend was observed for the Dose RP, which was significantly lower than all the available literature references. Furthermore, the fluoroscopy time was comparable to or shorter than that reported in most published studies. This discrepancy may reflect differences in radiation geometry, field size selection, the use of magnification, and dose optimization strategies. A more homogeneous distribution of radiation over a wider field may result in lower doses at the reference point, despite similar DAP values.

With regard to injection procedures, the details of which are included in Table 11, the fluoroscopy time was significantly shorter than that reported in most published studies, while the DAP dose and the dose at the reference point remained at comparable levels. This finding suggests that radiation exposure was not primarily due to prolonged fluoroscopic guidance, but rather to the limited number of images required to verify the procedure. Shorter fluoroscopy times may also reflect the operator’s increased experience, an optimized workflow, and the use of modern imaging technology, allowing for more effective target localization without compromising the performance of the procedure.

It should be emphasized that a key term in the European Directive definition of DRLs is the concept of a “typical examination”. This concept works well for straightforward imaging procedures such as chest, abdominal, or limb X-rays, where the examination type is clearly defined and consistent. However, it is less suitable for interventional procedures, which are often non-standard and may involve unexpected complications due to their complex nature. Since these procedures are performed for treatment rather than diagnosis alone, they are often open-ended and continue until the therapeutic goal is achieved [15].

Of special interest is the comparison of fluoroscopy DAP to cine DAP. The prevalence of fluoroscopic guidance is related to efforts to limit repeated cine acquisitions, given that cine mode is typically associated with a higher dose per unit of time [23,29,30]. Conversely, increased use of cine imaging may reflect a greater emphasis on high-resolution image recording and more detailed justification of interventions. The necessity for cine imaging depends on the type of invasive procedure being performed, taking into account the anatomical region; for example, there may be highly rapid blood flow (i.e., DSA cerebral) or precise mapping may be required to locate the structure of interest (i.e., embolization, TIPS and TACE). On the other hand, nephrostomy, vertebroplasty and injections rarely use cine acquisition; they track slow-moving tools or more static targets rather than fast blood flow. These findings demonstrate that the total radiation exposure does not depend exclusively on the duration of fluoroscopy but also on the relative contribution of the different imaging modalities during the procedure.

Beyond the comparison with previously published studies, the updated institutional DRLs established in the present work have important practical implications. They may support routine quality assurance activities, facilitate periodic review of imaging protocols, assist in identifying procedures with unexpectedly high radiation exposure, and provide locally relevant benchmarks for optimization initiatives. Furthermore, these institutional reference levels may contribute to future multicenter surveys and the development or revision of national DRLs by providing contemporary data obtained with modern angiography equipment under routine clinical conditions.

This study has several limitations that should be acknowledged. First, it was conducted at a single tertiary institution, which may limit the generalizability of the findings. Second, because of its retrospective design, patient age, body mass index, body habitus, procedural indication, and other anthropometric characteristics were not consistently available and therefore could not be included in the analysis. In addition, information regarding procedural complexity, operator experience, protocol modifications, and changes in clinical practice over the approximately ten-year interval was unavailable. These factors are recognized as determinants of radiation exposure in interventional radiology and may have contributed to the observed differences between the two study periods. Consequently, the findings should be interpreted as reflecting the combined influence of technological developments and multiple clinical factors rather than the isolated effect of the angiography equipment. Future prospective multicenter studies incorporating these variables would further strengthen the establishment and validation of institutional and national DRLs.

## 5. Conclusions

A key strength of this study is that the comparison was conducted within the same hospital environment, thereby minimizing inter-institutional variability associated with differences in clinical practice, imaging equipment, and heterogeneity of protocols. This allowed for a more reliable assessment of the impact of the technological upgrade on radiation exposure parameters. Furthermore, since the analysis was based on real-world clinical data collected during daily routine, the findings provide important insights into the practical performance of modern fluoroscopy systems under real-world working conditions.

In summary, the changes observed following the transition to the newer fluoroscopy system underscore the important role of continuous technological advancement, the periodic reassessment of imaging protocols, and the systematic monitoring of radiation dose indices in optimizing radiation protection for both patients and healthcare personnel.

The present study provides updated institutional Diagnostic Reference Levels for a broad spectrum of interventional radiology procedures performed using contemporary angiographic equipment. The comparison with data collected approximately one decade earlier demonstrates that radiation exposure metrics evolve over time and are likely influenced by a combination of technological developments, changes in clinical practice, procedural complexity, and other factors that could not be fully quantified in this retrospective analysis. The updated local DRLs constitute a valuable tool for protocol optimization, quality assurance, and radiation dose monitoring, while also providing a useful benchmark for future multicenter investigations and the establishment of national Diagnostic Reference Levels.

## Figures and Tables

**Table 1:** Patient sex distribution and number of interventional radiology procedures performed using the two angiography systems.

	INFX-8000V—Type S	Azurion 7 B20/15
Female (%)	52.4	51.3
Male (%)	47.6	48.7
Number of Procedures		
DSA Cerebral	37	35
Embolization	49	42
Nephrostomy	39	45
Vertebroplasty	35	43
TIPS	39	38
TACE	40	36
Injection	39	45

**Table 2:** Technical specifications of radiography systems [18,19].

Feature	INFX-8000V—Type S	Azurion 7 B20/15
Generated Power (Maximum nominal electric power)	100 kW	100 kW
Focal Spot Size	0.3/0.6/1.0 mm (Frontal) 0.5/0.5/0.8 mm (Lateral)	0.4/0.7 mm (Frontal) 0.5/0.8 mm (Lateral)
Anode Heat Storage	3 MHU	6.4 MHU
Maximum Cooling Rate	7700 HU/s (462 kHU/min)	1750 kHU/min
Flat Panel Detector (Maximum X-ray field)	FPD8: 20 cm × 20 cm FPD12: 30 cm × 30 cm FPD1216: 30 cm × 40 cm	24.5 × 24.5 cm (SID = 70) 35 × 35 cm (SID = 100) 42 × 42 cm (SID = 120)
Fluoroscopy Pulse Rates	Continuous, 1, 2, 3, 5, 7.5, 10, 15, 20, 30 pps (can be selected at the time of installation)	Default at 3.75, 7.5, 12.5 and 30 pps
Pixel Pitch	194 µm × 194 µm	184 μm × 184 μm
Resolution	2.6 lp/mm	2.72 lp/mm
DQE (0)	65% at 0 lp/mm	70% at 0 lp/mm

**Table 3:** Descriptive statistics for DAP values.

	DAP (Gy∙cm^2^)										
Procedure	Mean		25th		Median		Relative Difference	75th		IQR	
	Old	New	Old	New	Old	New		Old	New	Old	New
DSA Cerebral	56.83	21.22	22.20	10.32	36.40	16.35	−55.1%	86.65	25.15	64.45	14.83
Embolization	184.83	282.13	19.38	131.84	86.17	200.43	+132.6%	359.44	405.89	340.06	274.05
Nephrostomy	15.70	9.85	2.53	2.75	4.93	5.72	+16%	23.64	12.49	21.11	9.74
Vertebroplasty	132.37	136.59	73.90	78.47	87.39	113.73	+30.1%	185.50	180.26	111.60	101.79
TIPS	197.79	366.17	64.05	152.33	166.39	349.60	+110.1%	309.35	508.26	245.30	355.94
TACE	165.94	313.03	52.65	114.46	93.73	202.49	+116.0%	156.81	523.58	104.16	409.12
Injection	14.62	28.05	3.14	10.86	6.79	18.56	+173.3%	14.67	32.70	11.53	21.85

**Table 4:** Descriptive statistics of total dose at Reference Point (RP).

	Dose RP total (mGy)										
Procedure	Mean		25th		Median		Relative	75th		IQR	
	Old	New	Old	New	Old	New	Difference	Old	New	Old	New
DSA Cerebral	277.4	161.4	70.3	75.5	208.3	106.6	−48.8%	402.4	210.1	332.1	134.6
Embolization	1081.1	885.9	109.0	334.3	782.1	664.4	−15.0%	1613.9	1107.1	1504.8	772.8
Nephrostomy	145.8	70.7	16.8	19.0	77.2	33.7	−56.4%	164.7	86.9	147.9	67.8
Vertebroplasty	1715.6	1275.4	559.4	880.0	1325.9	1203.8	−9.2%	2242.0	1439.4	1682.6	559.3
TIPS	895.1	1331.9	260.2	514.9	808.9	1086.4	+34.3%	1524.7	1739.4	1264.5	1224.6
TACE	761.8	813.9	245.7	288.1	452.4	549.5	+21.5%	687.6	1549.8	441.9	1261.7
Injection	137.6	265.2	27.8	84.0	87.9	134.6	+53.1%	170.7	281.0	142.9	197.0

**Table 5:** Descriptive statistics of total fluoroscopy time.

	Fluoroscopy Time (min)										
Procedure	Mean		25th		Median		Relative	75th		IQR	
	Old	New	Old	New	Old	New	Difference	Old	New	Old	New
DSA Cerebral	9.6	2.8	5.6	1.5	7.2	2.2	−69.8%	13.0	3.7	7.4	2.2
Embolization	20.1	21.6	9.5	9.5	18.5	17.0	−8.1%	27.1	27.8	17.6	19.3
Nephrostomy	4.3	3.9	2.0	2.3	3.3	3.0	−9.5%	5.2	5.0	3.2	2.7
Vertebroplasty	19.0	17.8	12.7	11.3	18.4	15.3	−16.8%	22.6	25.4	9.9	14.1
TIPS	17.9	32.9	9.6	14.3	16.4	26.3	+60.4%	26.5	51.8	16.9	37.4
TACE	15.3	20.5	8.4	12.5	12.6	20.1	+60.3%	17.6	28.6	9.2	16.1
Injection	2.6	4.4	1.1	2.0	2.1	2.8	+32.3%	3.1	4.1	2.0	2.2

**Table 6:** Mann–Whitney U test results.

Procedure	DAP			Total Time		
	U	*p*-Value	Effect Size (r)	U	*p*-Value	Effect Size (r)
DSA Cerebral	42	0.001	0.59	77	0.004	0.44
Embolization	0	0.001	0.84	3	0.001	0.83
Injection	181	0.002	0.40	241	0.042	0.28

**Table 7:** Comparison of studied parameters for DSA procedures [20,22,24].

DSA			
	DAP (Gy·cm^2^)	Dose RP Total (mGy)	Time (min)
This study	76.35	106.6	2.2
Slare et al. [20]	157.50	598.5	18.8
Papanastasiou et al. [20]	50.40	367.5	5.2
Actan et al. [20]	74.00	-	-
Tristram et al. [20]	30.80	195.0	5.3
Sowa et al. [24]	6.59–157.50	36.7–598.5	2.5–18.8
Opitz et al. [22]	214.19	-	17.2–117.2

**Table 8:** Comparison of studied parameters for embolization procedures [20,26].

Embolization			
	DAP (Gy·cm^2^)	Dose RP Total (mGy)	Time (min)
This study	200.40	664.4	17.0
Inh et al. [26]	130.60	2104.0	40.9
Hassau et al. [26]	78.70	1040.0	25.7
Tristam et al. [20]	132.80	1397.0	51.4
Aly et al. [26]	85.00	801.0	19.5
Rizk et al. [26]	91.00	1113.0	15.0
Sowa et al. [26]	13.71	212.5	20.4
Actan H. et al. [20]	259.00	-	-

**Table 9:** Comparison of studied parameters for TIPS procedures [27,28].

TIPS			
	DAP (Gy·cm^2^)	Dose RP Total (mGy)	Time (min)
This study	349.60	1086.4	26.3
Pimenta et al. [27]	312.00	1367.7	32.1
Etard et al. [27]	185.00	780.0	39.0
Compagnone et al. [28]	337.56	-	18.1

**Table 10:** Comparison of studied parameters for TACE procedures [27].

TACE			
	DAP (Gy·cm^2^)	Dose RP Total (mGy)	Time (min)
This study	202.49	549.5	20.1
Pimenta et al. [27]	247.70	1130.7	20.5
RP 195 [27]	241.00	1867.0	18.0
Schegerer et al. [27]	224.00	-	25.0
Rizk et al. [27]	522.00	2890.0	29.0
Etard et al. [27]	250.00	990.0	28.0
Ruiz-Cruces et al. [27]	303.00	-	26.3
Compagnone et al. [27]	177.70	-	16.2

**Table 11:** Comparison of studied parameters for injection procedures [27].

Injections			
	DAP (Gy·cm^2^)	Dose RP Total (mGy)	Time (min)
This study	18.60	131.6	2.8
Pimenta et al. [27]	20.20	148.8	7.2
RP 195 [27]	22.00	194.0	10.0
Rizk et al. [27]	148.00	990.0	20.0
Etard et al. [27]	35.00	260.0	16.0
Ruiz-Cruces et al. [27]	30.00	-	17.3

## Data Availability

The original contributions presented in this study are included in the article. Further inquiries can be directed to the corresponding author.
